# Co-expression network-based identification of biomarkers correlated with the lymph node metastasis of patients with head and neck squamous cell carcinoma

**DOI:** 10.1042/BSR20194067

**Published:** 2020-02-20

**Authors:** Yu Jin, Xing Qin

**Affiliations:** 1 Department of General Dentistry, Ninth People’s Hospital, Shanghai Jiao Tong University School of Medicine, Shanghai 200011, P.R. China; 2 Shanghai Key Laboratory of Stomatology and Shanghai Research Institute of Stomatology, National Clinical Research Center of Stomatology 200000, P.R. China; 3 Department of Oral and Maxillofacial-Head and Neck Oncology, Ninth People’s Hospital, Shanghai Jiao Tong University School of Medicine, Shanghai 200011, P.R. China

**Keywords:** biomarker, Gene Expression Omnibus (GEO), gene set enrichment analysis (GSEA), head and neck squamous cell carcinoma, prognosis, weighted gene coexpression network analysis (WGCNA)

## Abstract

Head and neck squamous cell carcinoma (HNSCC) is ranked as one of the most frequent malignancies worldwide with a high risk of lymph node metastasis, which serves as a main reason for cancer deaths. Identification of the potential biomarkers for lymph node metastasis in HNSCC patients may contribute to personalized treatment and better therapeutic effect. In the present study, GSE30788 microarray data and corresponding clinical parameters were downloaded from Gene Expression Omnibus (GEO) and Weighted Gene Co-expression Network Analysis (WGCNA) was performed to investigate significant modules associated with clinical traits. As a result, the genes in the blue module were determined as candidate genes related with HNSCC lymph node metastasis and ten hub genes were selected from the PPI network. Further analysis validated the close associations of hub gene expression with lymph node metastasis of HNSCC patients. Furthermore, survival analysis suggested the level of Loricrin (LOR) was statistically significantly associated with the disease-free survival of HNSCC patients, indicating the potential of utilizing it as prognosis predictor. Overall, our study conducted a co-expression network-based analysis to investigate significant genes underlying HNSCC metastasis, providing promising biomarkers and therapeutic targets.

## Introduction

Head and neck squamous cell carcinoma (HNSCC) is a common malignancy with high incidence and mortality. Surgery therapy, multidrug chemotherapy and local radiotherapy are the traditional and routine treatment methods for HNSCC. In spite of enormous improvement in diagnosis and treatment strategies, the survival rate of HNSCC patients still remains stagnant. Therefore, investigating the essential factors influencing HNSCC patient prognosis and inventing innovative therapies with high efficacy are of great importance.

It was widely established that highly invasive and metastatic abilities are the characteristic property of HNSCC. In most cases, HNSCC *in situ* may not be fatal to patients, while HNSCC with lymph node or distant metastasis could significantly impair patients’ life quality and even cause cancer-related deaths. It was reported that regional and distant metastasis constitutes a large proportion of HNSCC treatment failures. To be more specific, the 5-year survival rate of HNSCC patients with nodal metastasis dropped to 30% when compared with those patients without metastasis. Thus, exploration of relative accurate and reliable methods to predict HNSCC metastasis could contribute to the personalized treatment with better responses and improved prognosis for HNSCC patients. With the remarkable advancement of microarray and sequencing technologies, a great deal of genomic information has been investigated and accumulated, which could facilitate cancer research [1]. Besides the development of new biological research technology, novel bioinformatics and data mining algorithms have also been invented to analyze transcriptomic data for the discovery of more precise and reliable biomarkers. Tumorigenesis is a complex process that includes multiple steps of transformation, including intricate interactions between genes and similar gene expression patterns. Weighted Gene Co-expression Network Analysis (WGCNA) is an innovative approach to quantitatively assess the internal connectivity of gene clusters inside the comprehensive network and evaluated the correlations of gene modules with clinical features [2]. It benefits to look into the molecular regulatory mechanisms in HNSCC and discovered potential crucial cancer-related genes. Biomarkers are defined as specific genes whose expression level could indicate a particular disease state. Until now, a variety of biomarkers have been identified and put into clinical practice, such as early diagnosis, prediction of therapeutic effect and prognosis evaluation. Therefore, identification of efficient biomarkers for early detection and prognosis evaluation of HNSCC patients might be of clinical significance [3].

Multiple studies concentrated on diverse cancers have applied WGCNA to investigate the significant genes which have close associations with clinical parameters. For example, five hub genes were identified to be promising predictors for breast cancer distant metastasis using WGCNA and PPI analyses [4]. Similarly, another study developed a robust mRNA signature which could be utilized as an independent predictor for lymph node metastasis in lung adenocarcinoma patients, laying a foundation for personalized treatment methods [5]. In osteosarcoma, an eight-gene signature was screened out to distinguish different metastasis state of cancer patients and made prognosis evaluation through integrating genome data and clinical information [6]. What is more, a study illustrated some candidate biomarkers which were closely related with TNM stage and the survival time of bladder cancer patients, serving as possible diagnostic markers or therapeutic targets [7].

In the present study, we conducted a co-expression network-based analysis on GSE30788 to investigate significant genes underlying HNSCC metastasis, providing promising biomarkers and therapeutic targets for HNSCC.

## Materials and methods

### Microarray data acquisition

GS‘E30788 dataset with a collection of genomic data and clinical information of HNSCC patients with or without lymph node metastasis was downloaded from Gene Expression Omnibus (GEO, https://www.ncbi.nlm.nih.gov/geo/) database. It was based on the GPL13953 platform containing a total of 222 HNSCC samples. The data after background correction and normalization were put into the WGCNA analysis.

### WGCNA co-expression network construction and significant module identification

‘WGCNA’ package in R was used to construct co-expression network of the whole gene expression matrix of GSE30788. First, sample clustering was performed to rule out outlier samples. Then, a soft threshold was determined based on scale independence and mean connectivity analysis. Moreover, a cluster dendrogram among modules and an eigengene adjacency heatmap between modules were generated. For the relationship of gene modules with clinical traits, the Pearson test was applied to identify clinically significant modules. The gene module statistically significant associated with lymph node metastasis was selected as the module of interest to undergo further analysis.

### PPI network construction and hub gene detection

All the genes in the blue module were put into the STRING (http://www.string-db.org/) database to calculate the innate connectivity of genes and then a network was established in Cytoscape. Statistically significant gene modules and hub genes were selected by the MCODE and cytoHubba plugin, respectively. Hub genes were determined as a set of genes with the highest connectivity in the PPI network.

### Association of hub gene expression with lymph node metastasis of HNSCC patients

UCSC Xena (http://xenabrowser.net/) is an online database from which users have access to genomic data and clinical information of different kinds of tumor samples, so as to investigate the potential correlations between specific gene expression and phenotypic variables such as TNM stage and patient prognosis. In the current study, we detected the relationship of hub gene expression with the lymph node metastasis of HNSCC patients based on the TCGA data.

### Survival analysis

GEPIA (http://gepia.cancer-pku.cn/) is an online tool based on TCGA data from which patient survival analysis could be performed. In the present study, we evaluated the impact of hub gene expression on the survival time of HNSCC patients to investigate whether they could be used as effective prognosis predictors.

### Gene set enrichment analysis

In order to have an in-depth understanding of the underlying mechanisms of selected genes in HNSCC progression, Gene set enrichment analysis (GSEA) was implemented for GO and KEGG enrichment analysis. More specifically, HNSCC samples were divided into two groups according to the median level of Loricrin (LOR). Then, GSEA was performed and statistically significant results were identified.

### Statistical analysis

Statistical analysis was carried out using IBM SPSS STATISTICS 23.0 (International Business Machines, U.S.A.) throughout the present study. Comparisons between two groups were performed using non-parametric Mann–Whitney U test. Kaplan–Meier method was utilized to analyze the associations of hub gene with HNSCC patient survival based on the logrank test. *P*-value <0.05 was considered statistically significant.

## Results

### Co-expression module construction and significant module identification

**Figure 1:**
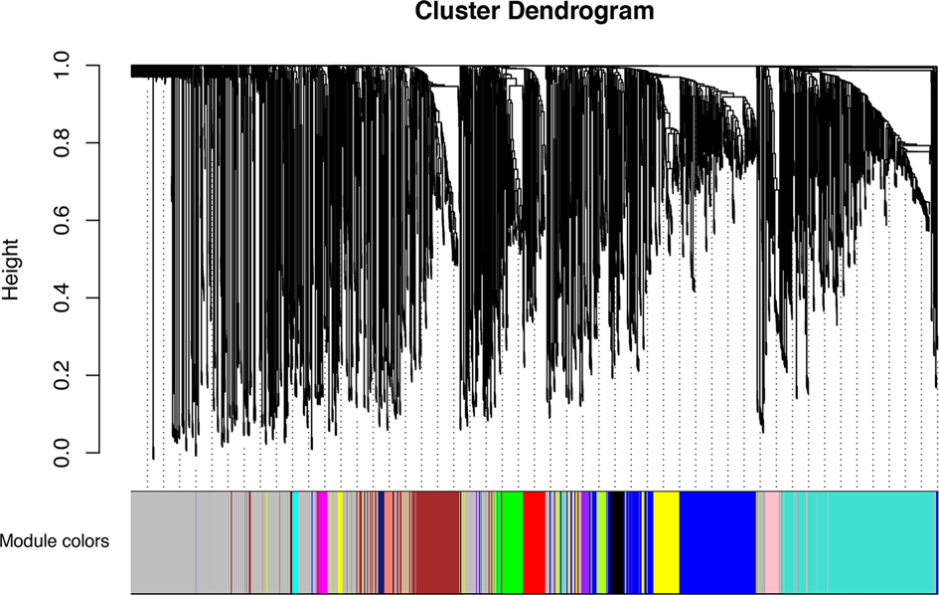
The cluster dendrogram of all the genes in GSE30788 Each branch represents one individual gene, and each color below represents one co-expression gene module.

**Figure 2:**
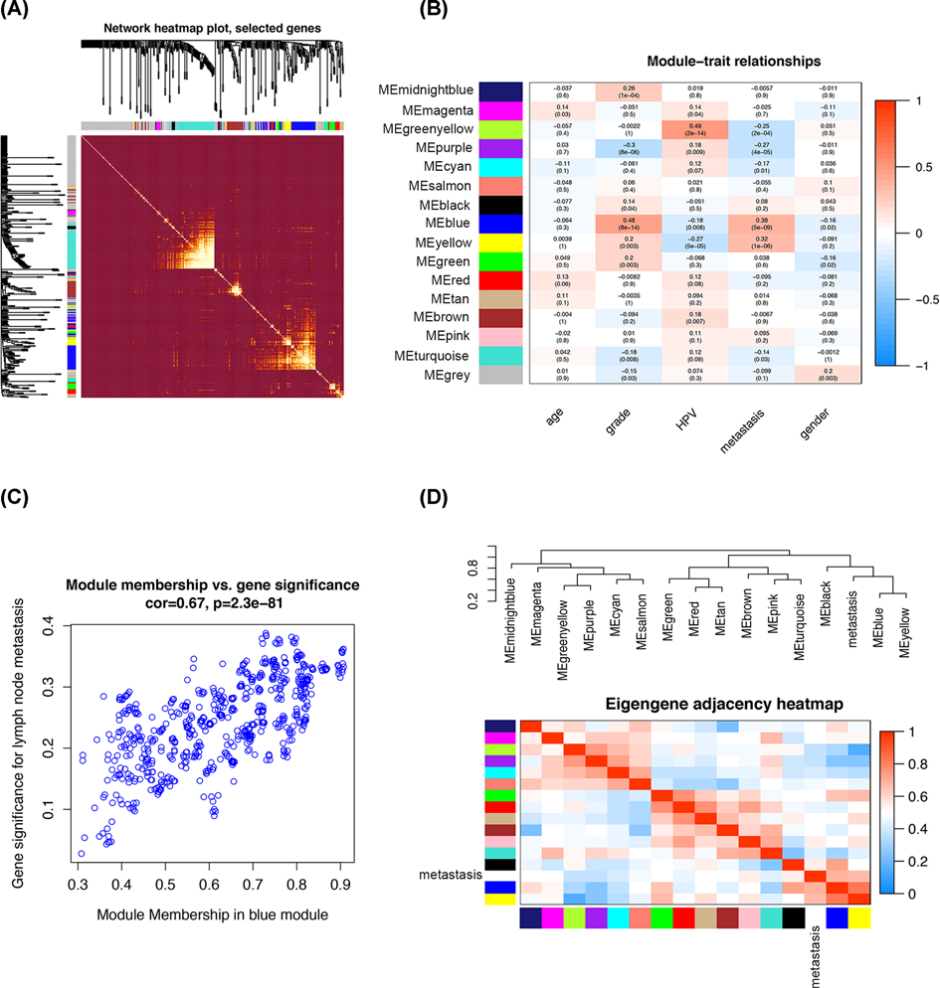
Module–trait relationships (**A**) Analysis of the interaction relationship of co-expression genes. (**B**) Heatmap of the correlation between module eigengenes and the clinical features. Corresponding correlation and *P*-value was presented, respectively. (**C**) Scatter diagram for correlation between module membership and gene significance of lymph node metastasis in the blue module. (**D**) Heatmap plot of the adjacencies in the hub gene network.

In the present study, we obtained the expression matrix of 222 HNSCC samples in GSE30788 and conducted WGCNA analysis. Specifically, the HNSCC samples included 154 males and 68 females. Among the patients, 75 people were younger than 65 years, 54 people were 65 years or older. Sample cluster was performed using Pearson’s correlation analysis to filter outlier samples. We selected the power of β = 6 as the soft-thresholding to construct a matrix of similarity of all pairs of genes. Then, a variety of gene modules were screened out by the means of average linkage hierarchical clustering (Figure 1). A heatmap of the interactive relations of the gene modules was plotted (Figure 2A). With the purpose to figure out genes which have clinical significance, we further conducted statistical analysis to identify modules closely related to clinical features. It turned out that the module eigengene of the blue module showed the highest connectivity with lymph node metastasis in HNSCC patients (Figure 2B). The correlation between module membership and gene significance in blue module was illustrated in Figure 2C. Finally, we calculated and clustered eigengenes based on their correlations to further explore co-expression similarity and the results were manifested in Figure 2D.

### Hub gene identification via the PPI network

**Figure 3:**
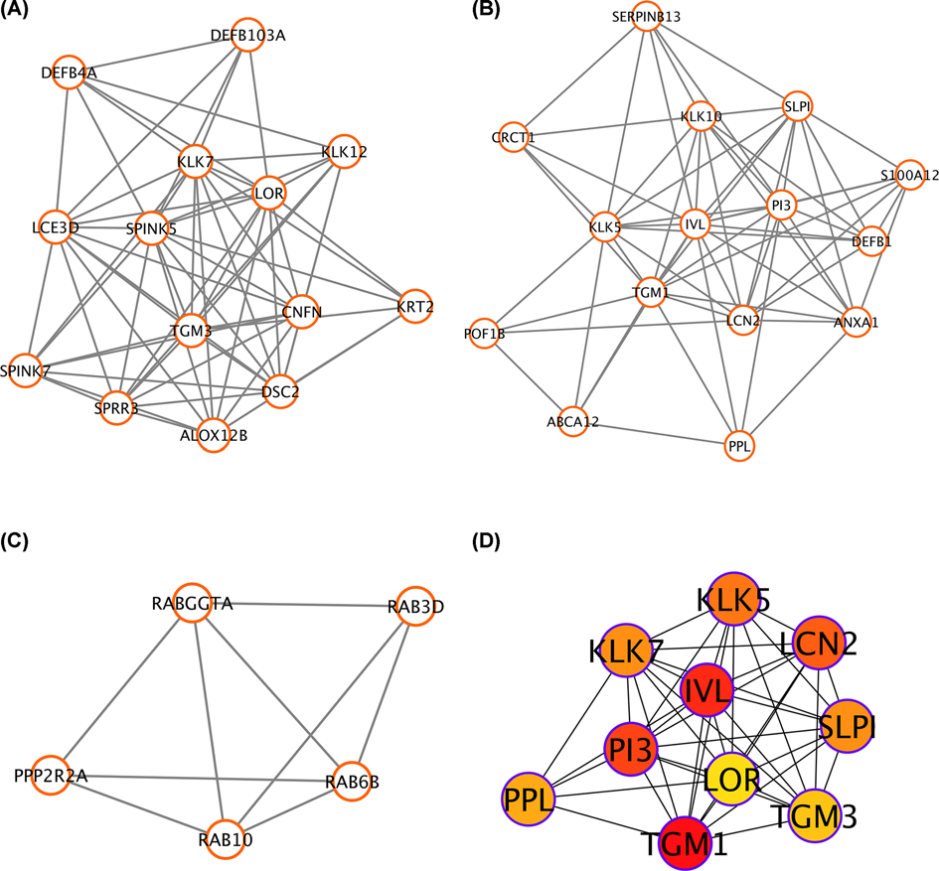
Significant gene modules and hub gene identification (**A**–**C**) Three most significant gene modules were screened out by the MCODE plugin in Cytoscape. (**D**) Ten hub genes were selected by the cytoHubba plugin in Cytoscape.

In the present study, we selected blue module as most significantly associated with lymph node metastasis in HNSCC patients and constructed a comprehensive PPI network. Subsequently, the top three significant modules were identified by the MCODE software and the cytoHubba tool was used to screen out hub genes based on the connectivity degree (Figure 3A–C). As a result, the top ten genes (Transglutaminase-1 (*TGM1*), Involucrin (*IVL*), *PI3*, Lipocalin-2 (*LCN2*), Kallikrein gene 5 (*KLK5*), secretory leukocyte protease inhibitor (*SLPI*), Kallikrein-related peptidase 7 (*KLK7*), Periplakin (*PPL*), Transglutaminase-3 (*TGM3*), *LOR*) exhibiting the highest connections with other genes were identified for further investigation (Figure 3D).

### Validation of hub gene on TCGA data

**Figure 4:**
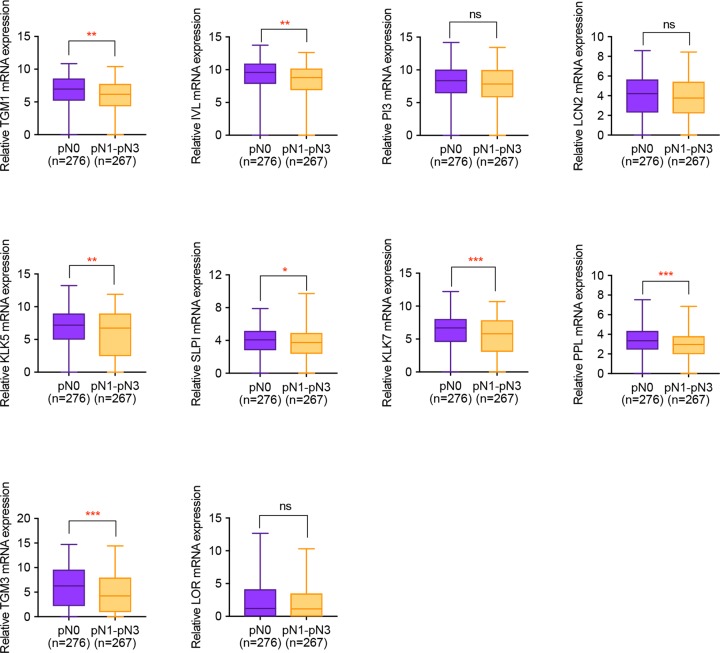
The correlation of hub gene expression with clinical lymph node metastasis of HNSCC patients Non-parametric Mann–Whitney U test was used to make comparisons between two groups. *, *P*<0.05. **, *P*<0.01. ***, *P*<0.001.

**Figure 5:**
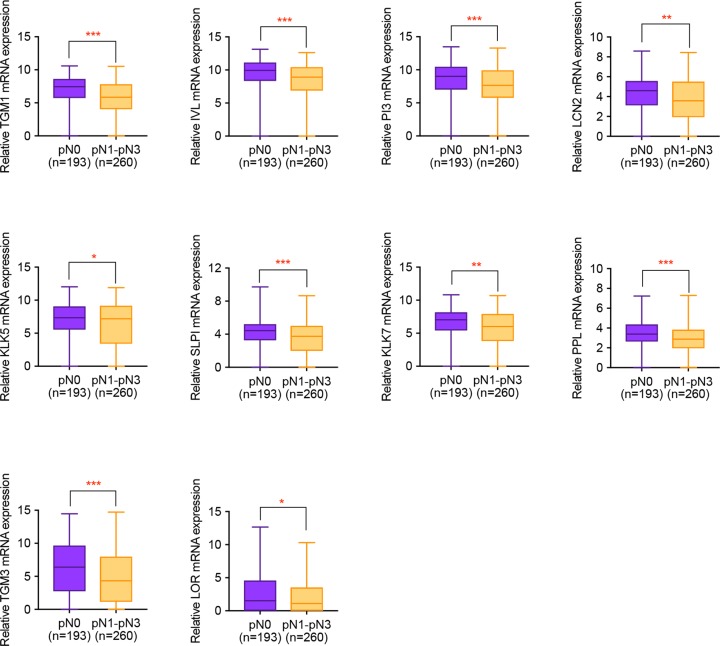
The relationship of hub gene expression with pathological lymph node metastasis of HNSCC patients Non-parametric Mann–Whitney U test was used to make comparisons between two groups. *, *P*<0.05. **, *P*<0.01. ***, *P*<0.001.

For further validation of the influences of hub gene on HNSCC lymph node metastasis, expression profiles of HNSCC patients with or without metastasis was downloaded from Xena database and made statistical analysis. It was concluded from Figure 4 that apart from *PI3, LCN2* and *LOR*, all the remaining hub genes presented remarkable differential expression in HNSCC samples with or without clinical lymph node metastasis. Meanwhile, based on the pathological diagnosis of lymph node metastasis, all the hub genes were statistically significant, implying the importance of hub gene in HNSCC progression (Figure 5).

### Survival analysis of hub gene

**Figure 6:**
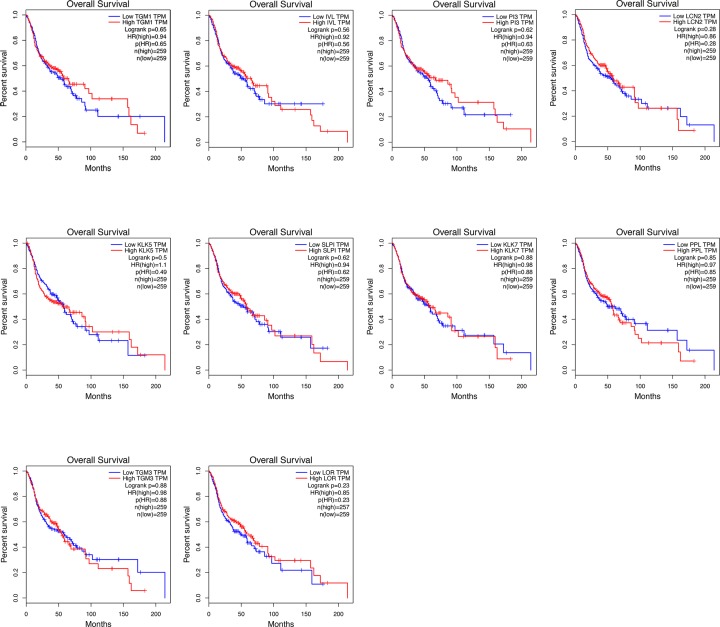
The influence of hub gene level on the overall survival time of HNSCC patients Kaplan–Meier method was utilized to make survival analysis.

**Figure 7:**
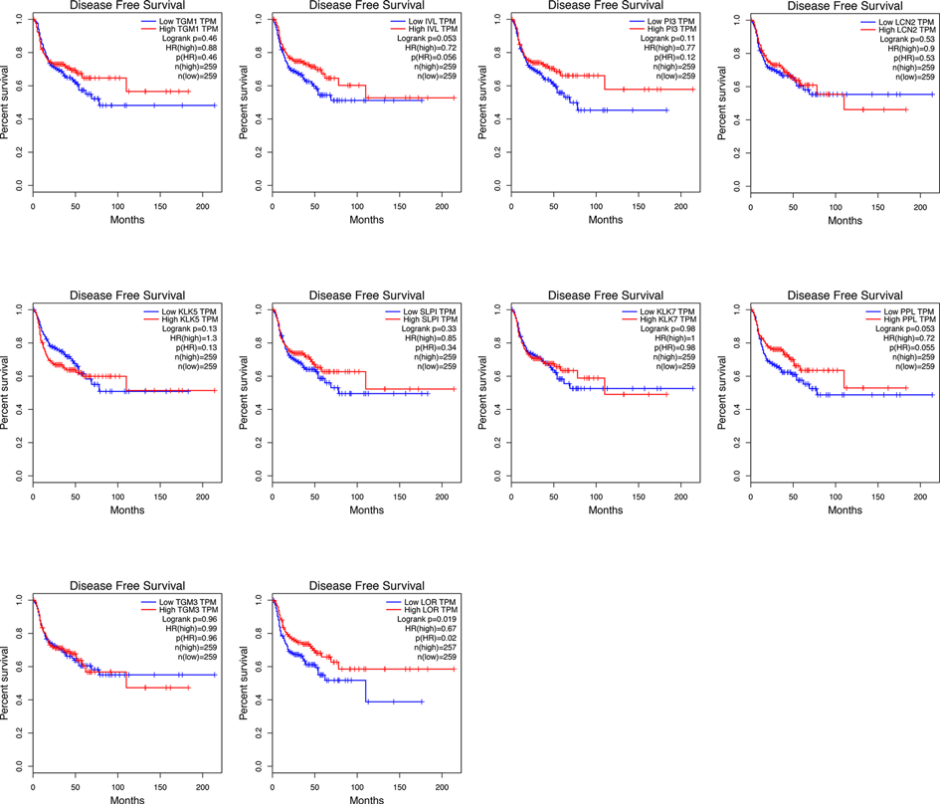
The effect of hub gene level on the disease-free survival time of HNSCC patients Kaplan–Meier method was utilized to make survival analysis.

To investigate the prognostic value of hub gene on HNSCC patients, survival analyses were performed using TCGA data obtained from GEPIA database. As was shown in Figure 6, all the genes had no evident impact on the overall survival time of HNSCC patients. While the analysis results of disease-free survival time suggested the down-regulation of LOR led to a poor prognosis of HNSCC patients (Figure 7), implying the possible biological functions of LOR on HNSCC pathogenesis.

### GSEA

**Figure 8:**
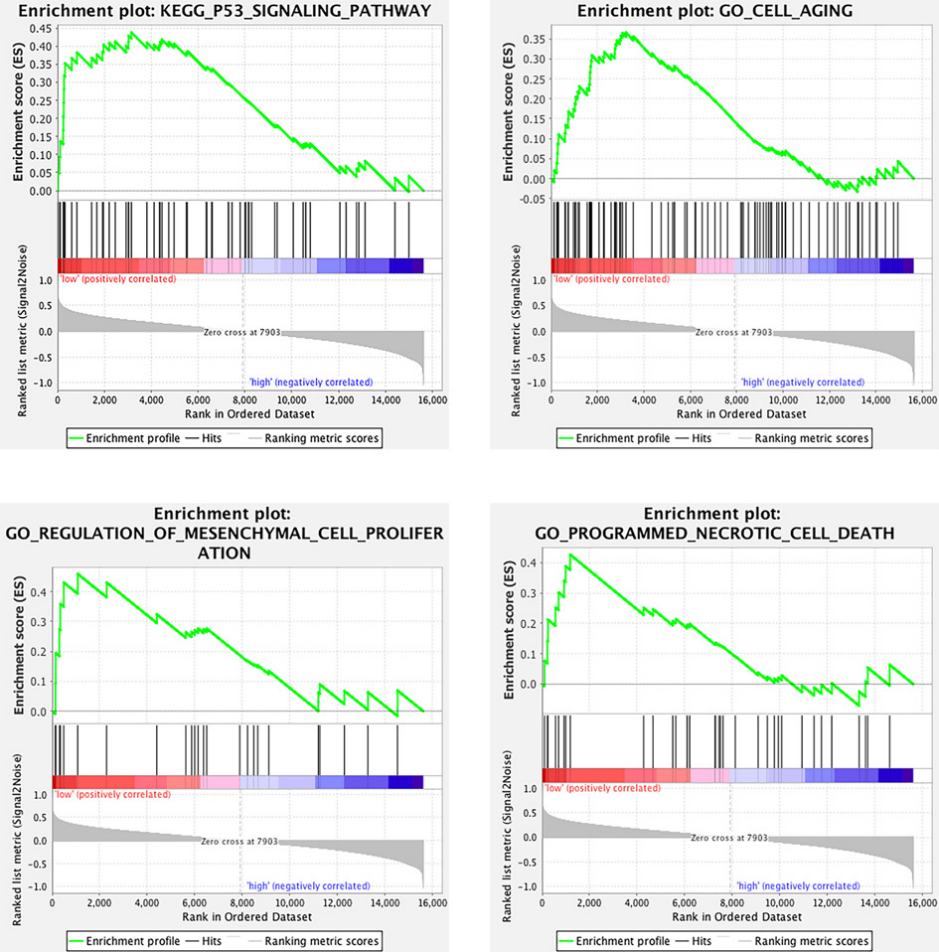
GSEA for expression profiles in HNSCC samples with relative lower expression of LOR Statistical significance was determined as *P*<0.05.

To further validate the functions of LOR in HNSCC, GSEA was conducted to identify GO and KEGG pathways enriched in HNSCC samples with relative lower expression of LOR. As was shown in Figure 8, gene set of ‘P53 signaling pathway’, ‘cell aging’, ‘regulation of mesenchymal cell proliferation’ and ‘programmed necrotic cell death’ were screened out to be statistically significant.

## Discussion

Carcinogenesis is a complex process during which a multiple of gene expression may be altered, allowing cells to escape homeostasis and experience a series of biological processes [8]. Transcription level regulation has been illustrated to play crucial roles in gene expression, implying the importance of utilizing microarray data conducted on a variety of cancers. HNSCC is a malignancy characterized by frequent lymph node metastasis. Since local and distant metastasis account for a majority of deaths in HNSCC patients, detection of effective markers for predicting lymph node metastasis in HNSCC is greatly needed.

WGCNA is a newly invented analysis method through which gene co-expression network could be constructed and significant modules could be identified for presenting insights into cancer biology [9]. Compared with other analysis methods, WGCNA hierarchical clustering method focused on the whole genome information instead of a previous selection of genes, which could avoid bias and subject decisions. It provides an overview of the signature of gene networks in cancer pathogenesis at system level. So far, a multiple of studies have utilized WGCNA to detect crucial gene modules correlated with clinical parameters so as to lay a groundwork for future applications. For example, a study revealed colon cancer recurrence-associated genes by WGCNA analysis, which may contribute to the development of novel therapies for preventing tumor recurrence [10]. Another study explored the potential regulatory mechanism of breast cancer progression based on WGCNA, mainly by identifying key KEGG pathways and constructing the comprehensive network of related transcription factors and microRNAs [11]. Also, a co-expression network was constructed in osteosarcoma to unravel the candidate gene clusters involved in tumor pathogenesis so as to seek promising therapeutic targets [12]. Moreover, a study applied this integrated bioinformatics approach to present gene modules significantly associated with prostate cancer grade, identifying potential diagnostic markers and prognosis indicators [13]. What is more, by using WGCNA method, a study invented a five-gene based prognosis predictor for breast cancer, providing basic foundation for future clinical research [14].

In the current study, after a series of data preprocessing, gene co-expression construction and significant module identification, we finally figured out one gene module which may be closely associated with the lymph node metastasis of HNSCC patients. Candidate genes were subjected to construct a comprehensive PPI network and ten hub genes (*TGM1, IVL, PI3, LCN2, KLK5, SLPI, KLK7, PPL, TGM3, LOR*) were finally identified, suggesting their potential functions on HNSCC metastasis. TGM1, which belongs to the transglutaminases (TGM) family, was demonstrated to be involved in cancer initiation and progression. *In vitro* experiments indicated the biological effect of TGM1 on gastric cancer cell proliferation and apoptosis. Furthermore, the regulation of TGM1 also influenced cancer cell sensitivity to chemotherapeutic drugs and the underlying mechanism may involve Wnt signaling pathway [15]. IVL has been well established as a highly specific and sensitive marker for cell differentiation and illustrated to present differential expression in distinct stages of cancers. More specifically, the immunohistochemical staining of IVL in cervical carcinoma *in situ* was remarkably lower than that in invasive cervical tumors [16]. For dysplastic leukoplakia, mild to moderate dysplasia presented relatively higher IVL expression, compared with leukoplakia with severe dysplasia, implying the participation of IVL in oral tissue malignant transformation [17]. PI3K signaling is a widely acknowledged important regulatory pathway in cancer-related processes, such as cell growth, survival and proliferation [18]. Regarding the significance of PI3K signaling pathway in tumor pathogenesis, *PI3* gene may also play indispensable roles in carcinogenesis process. LCN2 is a glycoprotein of the lipocalin superfamily which could be secreted by adipocytes, immune cells and tumor cells and was suggested to be implicated in a variety of cancers. Elevated serum LCN2 levels have been observed in breast, ovarian, colorectal and gastric cancer, serving as efficient biomarker [19]. Furthermore, LCN2 was illustrated to be a vital regulator in EMT, invasion and metastasis of colorectal cancer, mainly manifested by its correlation with E-cadherin and β-catenin expression. Meanwhile, it could inhibit the translocation of NF-κB into nucleus, representing a promising therapeutic target for colorectal cancer [20]. More importantly, LCN2 was validated to mediate the communications between tumor stroma and tumor cells, stimulating the epithelial–mesenchymal transition process in breast cancer [21]. *KLK5* was a member of Kallikrein gene family and suggested to have close associations with tumorigenesis. To be more specific, KLK5 was exploited as an independent marker for early detection and prognosis evaluation in prostate cancer [22]. Also, the level of KLK5 was intimately related with clinical parameters such as tumor grade and disease stage in ovarian cancer. Positive correlation of KLK5 expression with increased risk for relapse and death in ovarian cancer was illustrated as well [23]. The SLPI is a single-chain protein originally identified as a serine proteinase inhibitor against neutrophil elastase, cathepsin G, trypsin and chymotrypsin. However, more and more researches have emphasized its effect on multiple cancers and some explored its involvement in tumor metastasis. A study showed that SLPI has the ability to decrease the liver-metastasizing potential of tumor cells and this effect may be attained by reducing the production of hepatic TNF-α and E-selectin [24]. Another study determined SLPI as a metastasis promoter and could be utilized as novel therapeutic target for anti-metastatic therapies in breast and colon cancers [25]. The opposite findings may due to the diverse kinds of tumor types and individual discrepancy. Given the detailed function of SLPI on the lymph node metastasis of HNSCC, more comprehensive investigation needs to be implemented. KLK7 was found to be aberrantly expressed in various cancers, such as breast cancer, lung carcinoma and colon cancer. Investigation focused on the functional mechanism underlying KLK7 suggested that it could cleave pro-MMP9-generating active MMP9 and induce the shedding of cell adhesion proteins so as to mediate cancer cell proliferation and invasion [26]. Clinically, patients with KLK7 positive tumors have poor prognosis, with both shorter overall survival time and disease-free survival time, indicating its candidate role as unfavorable prognosis marker [27]. PPL is a membrane-associated protein with functions in cellular movement and attachment. It was demonstrated that PPL acts as an essential cell cycle regulator that its loss of expression may lead to uncontrolled cell proliferation by reversing the G_0_/G_1_ phase arrest [28]. Moreover, the findings of previous study were consistent with ours that PPL was significantly down-regulated in HNSCC samples with lymph node metastasis when compared with lymph node negative tumors [29]. What is more, PPL was verified to be an effective biomarker for esophageal cancer early detection and tumor progression [30]. TGM3 has been reported to be relevant to many malignant phenotypes. It was illustrated that the immunohistochemical evaluation of TGM3 expression level could help to predict tumor recurrence and contribute to improve the treatment outcome of patients with attentive follow-up [31]. Furthermore, TGM3 silencing caused by DNA hypermethylation in HNSCC promoted cell growth and inhibited apoptosis activities, indicating its potential tumor suppressor role [32]. LOR dysregulation was regarded as an indicator in the early stages of potentially malignant disorders including oral submucous fibrosis and leukoplakia [33]. It functions as a protective barrier upon traumatic stimuli and served as an efficient prognostic marker [34]. The significance of LOR in oral premalignant lesions has been emphasized, further investigation on its possible roles in HNSCC is worthwhile to be conducted. From what has been discussed above, it came to the conclusion that all the hub genes we identified have been more or less previously reported to be associated with tumor progression, either as biomarkers for diagnosis and prognosis, or act as biological phenotypes regulator. Therefore, future in depth research deserves to be performed.

Since LOR from the hub genes was the only one that had prognostic value in HNSCC patients, GSEA was conducted to demonstrate the GO and KEGG pathways enriched in HNSCC samples with relative lower expression of LOR. Statistical results screened out the gene set of “P53 signaling pathway,’ ‘cell aging,’ ‘regulation of mesenchymal cell proliferation’ and ‘programmed necrotic cell death’, implying the possible functional roles of LOR in HNSCC. Overall, our findings may remarkably contribute to the understanding of molecular mechanisms underlying HNSCC so as to facilitate therapeutic decision-making, risk stratification and prognosis prediction for HNSCC patients.

Through the application of WGCNA analysis, our study not only validated the findings of former studies, but also investigated novel biomarkers for HNSCC metastasis. Hopefully, our research may help to develop personalized treatment for HNSCC patients, which may solve some individual cases with poor responses and prognosis. Although the present study made some innovative discovery of genes underlying lymph node metastasis, no experiments were conducted for validation, which is the limitation of our study. Therefore, *in vitro* and *in vivo* experiments remain to be implemented for investigating the detailed biological effect of candidate genes on HNSCC in the future. Moreover, larger cohorts of HNSCC patients with or without lymph node metastasis are needed to further validate the accuracy and reliability of using identified genes for biomarkers.

## Conclusion

In summary, we applied WGCNA approach to construct a gene co-expression network and identified one significant module (blue module) and ten hub genes (*TGM1, IVL, PI3, LCN2, KLK5, SLPI, KLK7, PPL, TGM3, LOR*), which were suggested to play crucial roles in the lymph node metastasis of HNSCC patients. The expression level of LOR was illustrated to be tightly related to the disease-free survival time of HNSCC patients, implying its promising role as prognosis indicator.

## Abbreviations

EMT: epithelial-mesenchymal transition
GSEA: Gene set enrichment analysis
HNSCC: head and neck squamous cell carcinoma
IVL: Involucrin
KLK5: Kallikrein gene 5
KLK7: Kallikrein-related peptidase 7
LCN2: Lipocalin-2
LOR: Loricrin
MMP9: Matrix metalloproteinase 9
PI3K: phosphatidylinositol 3-kinase
PPI: Protein-protein interaction
PPL: Periplakin
SLPI: secretory leukocyte protease inhibitor
TCGA: The Cancer Genome Altas
TGM1: Transglutaminase-1
TGM3: Transglutaminase-3
TNM: Tumor Node Metastasis
WGCNA: Weighted Gene Co-expression Network Analysis
